# A first-principles study of the switching mechanism in GeTe/InSbTe superlattices†

**DOI:** 10.1039/d0na00577k

**Published:** 2020-09-17

**Authors:** Chiara Ribaldone, Daniele Dragoni, Marco Bernasconi

**Affiliations:** Dipartimento di Scienza dei Materiali, Università di Milano-Bicocca Via R. Cozzi 55 I-20125 Milano Italy daniele.dragoni@unimib.it

## Abstract

Interfacial Phase Change Memories (iPCMs) based on (GeTe)_2_/Sb_2_Te_3_ superlattices have been proposed as an alternative candidate to conventional PCMs for the realization of memory devices with superior switching properties. The switching mechanism was proposed to involve a crystalline-to-crystalline structural transition associated with a rearrangement of the stacking sequence of the GeTe bilayers. Density functional theory (DFT) calculations showed that such rearrangement could be achieved by means of a two-step process with an activation barrier for the flipping of Ge and Te atoms which is sensitive to the biaxial strain acting on GeTe bilayers. Within this picture, strain-engineering of GeTe bilayers in the GeTe–chalcogenide superlattice can be exploited to further improve the iPCM switching performance. In this work, we study GeTe–InSbTe superlattices with different compositions by means of DFT, aiming at exploiting the large mismatch (3.8%) in the in-plane lattice parameter between GeTe and In_3_SbTe_2_ to reduce the activation barrier for the switching with respect to the (GeTe)_2_–Sb_2_Te_3_ superlattice.

## Abstract 2

*Via* density functional theory calculations, we devise a novel strain-engineered GeTe_3_/In_3_SbTe_2_ superlattice to reduce the power consumption of interfacial phase change memories.

## Graphical abstract

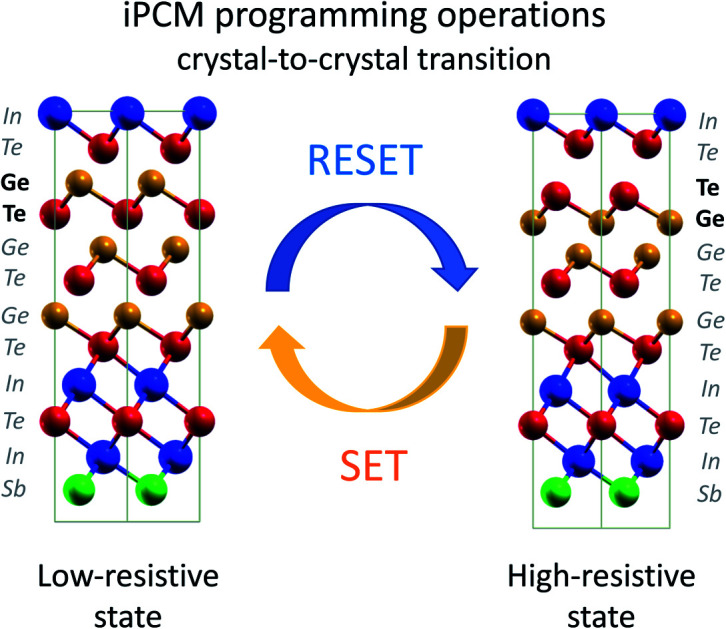

## 1 Introduction

Chalcogenide alloys are used as active materials in non-volatile phase change memories (PCMs) which rely on the reversible and fast transition between the amorphous and crystalline phases of the alloy induced by Joule heating.^1–6^ The two states of the memory can be discriminated thanks to a large difference in electrical resistivity between the two phases. The readout of the logical state consists of a measurement of resistance at low bias, while the SET/RESET operations require higher voltage pulses to induce either the melting of the crystal and subsequent amorphization or the recrystallization of the amorphous phase.

Alloys along the pseudobinary GeTe–Sb_2_Te_3_ tie-line such as the Ge_2_Sb_2_Te_5_ (GST) compound are commonly used because they provide a good compromise between transformation speed and data retention.

More recently, it has been shown that (GeTe)_*n*_(Sb_2_Te_3_)_*m*_ pseudobinary compounds arranged in superlattice (SL) geometries require a switching power in SET/RESET operations much lower than that needed in conventional GST alloys.^7^ In these devices, referred to as interfacial phase change memories (iPCMs),^7^ it was suggested that the transformation involves small displacements of a subset of atoms without melting and amorphization, the material remaining in a crystalline phase in both SET (low resistivity) and RESET (high resistivity) states.^7^ The SET state was proposed to correspond to a ferroelectric arrangement of the (GeTe)_2_ blocks (Ferro-GeTe) in the same geometry as that of the α phase of crystalline GeTe.^8^ The (GeTe)_2_ blocks are sandwiched by Sb_2_Te_3_ quintuple layers equal to those present in the bulk trigonal phase of Sb_2_Te_3_. The RESET state, on the other hand, was proposed^8^ to correspond to the so-called Inverted-Petrov structure ideally obtained by switching Ge and neighboring Te atoms in the crystalline structure of Ge_2_Sb_2_Te_5_ proposed by Petrov *et al.* (Petrov structure).^9^ Calculations based on Density Functional Theory (DFT) have indeed shown that the transformation between the Ferro-GeTe and Inverted-Petrov states can proceed *via* a two-step process consisting of a vertical flip of Ge atoms followed by a lateral motion.^10^ This transformation might possibly be induced by charge injection or electric fields.^11^

Furthermore, it has been proposed that the activation barriers for the SET/RESET transformations can be reduced by application of a biaxial strain to GeTe bilayers which can be achieved by the use of thicker Sb_2_Te_3_ blocks.^12^ Strain engineering could thus be exploited to further reduce power consumption for RESET/SET operations in iPCMs. An example of strain engineering control has also been reported for GeTe/Bi_2_Te_3_ heterostructures.^13^

This picture has, however, been brought into question by the Transmission Electron Microscopy (TEM), X-ray diffraction (XRD) and Extended X-ray Absorption Fine Structure (EXAFS) spectroscopy of SLs grown by molecular beam epitaxy (MBE).^14–18^ These measurements show that Ge atoms do not form GeTe bilayers but are instead incorporated within covalently bonded GeSbTe blocks of different sizes equal to the multiple layers present in the trigonal phase of crystals along the pseudobinary line (GeTe)_*n*_Sb_2_Te_3_. Sb atoms are mainly located closer to the van der Waals gap separating different (GeTe)_*n*_Sb_2_Te_3_ blocks, and intermixing of Ge/Sb in the same plane is observed. Similar conclusions have been drawn from the structural analysis of SLs grown by pulsed laser deposition (PLD)^19^ and magnetron sputtering.^20^ Still, the reduced power consumption for the SET/RESET transition is confirmed for SLs without GeTe blocks grown in ref. 14,15,19 and 20, which called for a reconsideration of the switching process in SLs.

On the basis of DFT calculations other mechanisms were then proposed involving the motion of the stacking fault^21^ or the inversion of SbTe bilayers leading to a reconfiguration of the van der Waals gaps which breaks the local chemical stoichiometry and results in a reversible metal–insulator transition.^22,23^ Experimental evidence for the inversion of SbTe bilayers has been reported in ref. 24. The ordering of randomly distributed vacancies into vacancy layers inside of Ge–Sb–Te building units has also been suggested as another switching mechanism.^25^ A very recent study suggests instead a more conventional switch due to thermally driven amorphization/crystallization in which the SL structure would allow for amorphization of stripes delimited by vdW gaps and for a better heat confinement responsible for the lower power consumption.^26^ Migration of voids in SLs under electric fields has also been proposed as a possible switching mechanism.^27^

In spite of all the different alternative models proposed so far, we might still conceive that the switching mechanism based on crystal-to-crystal transition between the Ferro-GeTe and Inverted-Petrov phases could occur in other SLs in which the replacement of the Sb_2_Te_3_ sub-blocks by other materials could prevent the incorporation of the GeTe blocks.

In this respect, we studied the geometry, the switching mechanism and the electronic structure of GeTe/In_3_SbTe_2_ SLs by means of DFT calculations. Actually, the three ternary compounds In_3_SbTe_2_ (IST), InGeTe_2_, and In_2_GeTe_3_ give rise to known crystalline phases, with the first and second ones also exploited as phase change materials.^28,29^ Stable crystals with compositions (GeTe)_*n*_SbInTe_3_ with *n* = 1–3 have also been synthesized in layered structures homologous to those of the (GeTe)_*n*_Sb_2_Te_3_ compounds.^30^ However, as soon as the concentration of indium is increased, as in the In_28_Ge_12_Sb_26_Te_34_ alloy studied in ref. 31, phase separation occurs during crystallization with the formation of crystalline In_3_SbTe_2_. This suggests indeed the possibility that GeTe might be less prone to being incorporated into cubic IST than into Sb_2_Te_3_ blocks.

The presence of GeTe bilayers in the relaxed configuration of GeTe/In_3_SbTe_2_ SLs allowed us to investigate the same switching mechanism proposed in ref. 10 for the (GeTe)_2_/Sb_2_Te_3_ SLs. Furthermore, we found that the large lattice mismatch between the In_3_SbTe_2_ and GeTe blocks can be exploited as a means to further reduce the activation barrier for SET/RESET transitions as proposed in ref. 12 for GeTe/Sb_2_Te_3_ SLs.

## 2 Computational details

The SLs have been studied by means of electronic structure calculations based on DFT and a plane wave expansion of Kohn–Sham (KS) orbitals as implemented in the Quantum-Espresso suite of programs.^32^ The Perdew–Burke–Ernzerhof (PBE) approximation^33^ to the exchange and correlation functional and norm conserving pseudopotentials with three, four, five and six valence electrons, respectively, for In, Ge, Sb and Te atoms were used. Geometry optimizations were performed by relaxing atomic positions and cell shape by using a Broyden–Fletcher–Goldfarb–Shanno quasi-Newton algorithm. To this end, a high energy cutoff of 75 Ry was used to minimize the Pulay stress. The energy barriers for the SET/RESET transitions were studied by using the Nudged Elastic Band (NEB) method with climbing image (CI) at fixed lattice parameters.^34^ These latter calculations were performed with a lower wavefunction cutoff of 40 Ry. Geometry optimizations and NEB calculations were performed by neglecting the spin–orbit (SO) interaction which was, however, used in the analysis of the electronic structure and in the estimate of the conductivity contrast between the SET/RESET states as described below.

## 3 Results

Before discussing the results on SLs, we briefly summarize the properties of bulk In_3_SbTe_2_ which is a constituent of our SLs. The IST compound is a phase change material considered in the past for applications in DVDs.^35^ Due to its high crystallization temperature, IST was also investigated for applications in PCMs which require data retention at temperature higher than those achievable with GST alloys.^28,36–42^ The crystal structure of cubic In_3_SbTe_2_ along the pseudobinary InSb–InTe tie-line was assigned to the *Fm*3̄*m* space group, with an experimental lattice constant of 6.126 Å.^41^ IST shares the rocksalt geometry of GeSbTe alloys in the metastable cubic phase with In atoms occupying the cationic sublattice and Sb and Te atoms randomly occupying the anionic sublattice, as shown by XRD and neutron diffraction experiments.^41,42^ Although crystalline IST is stable only in the temperature range of 555–435 °C, the metastable cubic phase can be recovered upon quenching under normal conditions, bypassing the decomposition into InTe and InSb.^43^ The IST crystal is metallic with an experimental electrical conductivity of 3.2 × 10^4^ S cm^−1^ under normal conditions.^42^

In previous DFT-PBE calculations,^44,45^ the rocksalt phase of IST was modeled in a hexagonal ordered cell with six planes stacked along the *c* axis as In–Sb–In–Te–In–Te and six atoms per unit cell. The optimization of the hexagonal cell by keeping the *c*/*a* ratio fixed to the value 
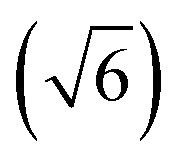
 expected for the rocksalt symmetry yielded a cubic lattice parameter of 6.181 Å which is only slightly larger than the experimental value of 6.126 Å.^44^

The six-layered unit cell of IST in the hexagonal geometry was then used as a sub-block in GeTe/IST superlattices with IST replacing the Sb_2_Te_3_ blocks of GeTe/Sb_2_Te_3_ SLs. Bilayers of GeTe were then stacked above IST along the SL growth axis which coincides with the *c* axis of IST in the hexagonal cell and with the *c* axis of trigonal α-GeTe in the hexagonal setting.

The theoretical in-plane equilibrium lattice parameter of bulk IST of *a*_hex_ = 4.37 Å is about 3.8% larger than the theoretical in-plane lattice parameter of α-GeTe^46^*a*_hex_ = 4.208 Å in the hexagonal setting (exp. 4.172 Å from ref. 47) as compared with the smaller lattice mismatch of 2.4% between bulk Sb_2_Te_3_ and α-GeTe.^12^ This feature suggests the possibility to reduce the activation barriers for the SET/RESET transitions in GeTe/IST with respect to GeTe/Sb_2_Te_3_ according to the arguments proposed in ref. 12.

We recall that the *a*_hex_ lattice parameter of trigonal α-GeTe can be obtained from the trigonal lattice parameters *a* and *α* using the relation *a*_hex_ = 2*a* sin(*α*/2).

### 3.1 Models of GeTe–InSbTe superlattices

We conceived the (GeTe)_*m*_/(In_3_SbTe_2_)_*n*_ superlattice as an alternation of six-layered In_3_SbTe_2_ blocks and (GeTe)_*m*_ blocks composed of *m* GeTe bilayers. The In_3_SbTe_2_ blocks were set with an In–Sb–In–Te–In–Te sequence, exposing In and Te atoms at the edges of the block. The hexagonal planes of the IST and GeTe blocks were all stacked in an ABCABC sequence implying the use of a hexagonal supercell when the number of atoms in the cell is a multiple of three or a trigonal supercell otherwise.

**Fig. 1:**
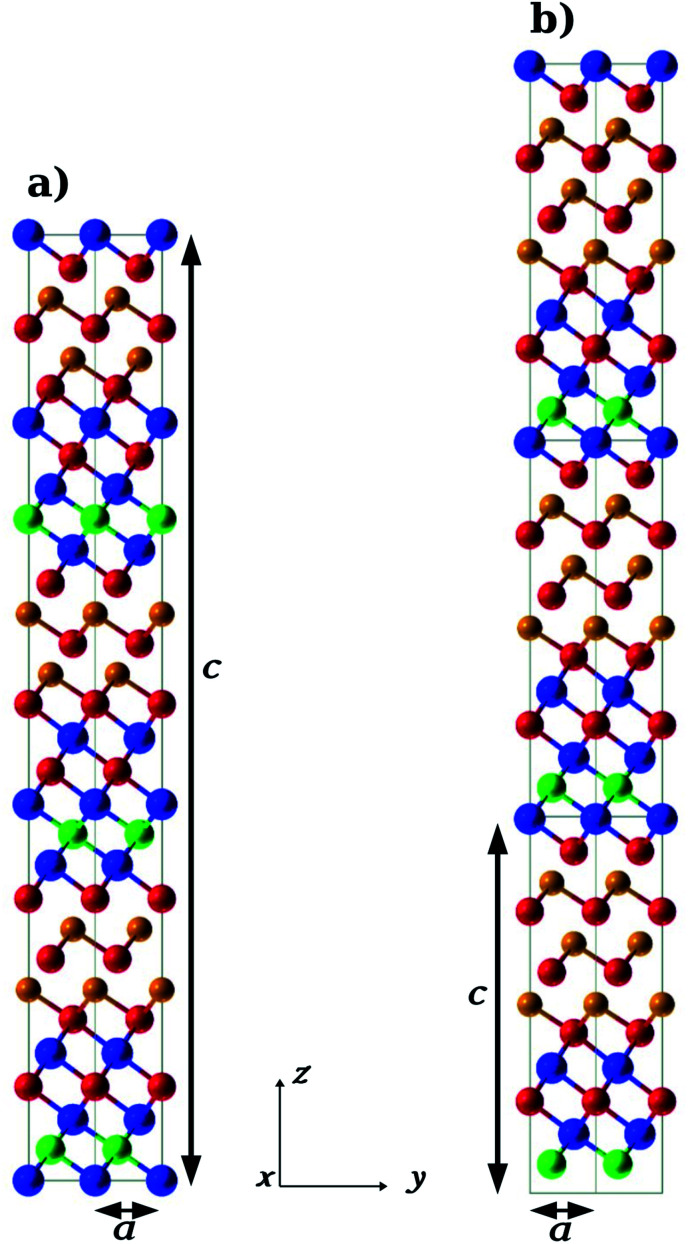
Equilibrium configuration of the (a) (GeTe)_2_/In_3_SbTe_2_ SL in the hexagonal setting (three formula units) and (b) (GeTe)_3_/In_3_SbTe_2_ SL. Three unit cells along the *c* axis are shown in panel (b) for the sake of comparison with panel (a). Red, orange, blue and green spheres correspond to Te, Ge, In and Sb atoms.

We optimized the geometry and lattice parameters of the (GeTe)_2_/In_3_SbTe_2_, (GeTe)_3_/In_3_SbTe_2_ and (GeTe)_3_/(In_3_SbTe_2_)_2_ SLs by integrating the Brillouin Zone (BZ) with a 20 × 20 × 20 Monkhorst–Pack (MP) mesh^48^ for the trigonal (GeTe)_2_/In_3_SbTe_2_ SL and an 18 × 18 × 6 mesh for the hexagonal (GeTe)_3_/In_3_SbTe_2_ and (GeTe)_3_/(In_3_SbTe_2_)_2_ SLs. The optimized geometries of (GeTe)_2_/In_3_SbTe_2_ and (GeTe)_3_/In_3_SbTe_2_ are shown in Fig. 1. The equilibrium lattice parameters of the SLs are compared with those of bulk In_3_SbTe_2_ and α-GeTe in the hexagonal setting in Table 1. Note that the conventional hexagonal cell of the (GeTe)_2_/In_3_SbTe_2_ SL is three times larger than the trigonal unit cell which contains a single formula unit. The atomic positions in crystal coordinates are given in Tables S1–S3 in the ESI.† Upon relaxation the two GeTe bilayers of (GeTe)_2_/In_3_SbTe_2_ undergo a reconstruction: a Ge layer binds to the outermost Te layer of the IST block while a Te layer binds to the outermost In layer of the IST. As a consequence only a GeTe bilayer survives. The same reconstruction takes place in the (GeTe)_3_/In_3_SbTe_2_ SL in which only two GeTe bilayers out of three survive in a configuration very similar to the Ferro-GeTe model of the (GeTe)_2_/Sb_2_Te_3_ SL.

**Table 1:** Lattice parameters of the optimized geometry of GeTe/IST superlattices and of bulk In_3_SbTe_2_ and GeTe, all in the hexagonal setting. The conventional hexagonal unit cell contains three formula units for the trigonal (GeTe)_2_/In_3_SbTe_2_ SL

Superlattice	*a* = *b* [Å]	*c* [Å]
(GeTe)_2_/In_3_SbTe_2_	4.307	52.859
(GeTe)_3_/In_3_SbTe_2_	4.293	21.141
(GeTe)_3_/(In_3_SbTe_2_)_2_	4.316	31.796
GeTe	4.208	10.749
In_3_SbTe_2_	4.370	10.644

The formation energy Δ*H* of the SLs with respect to the parent compounds GeTe and In_3_SbTe_2_ has been computed as follows:

1 Δ*H*[(GeTe)_*m*_/(In_3_SbTe_2_)_*n*_] = *E*[(GeTe)_*m*_/(In_3_SbTe_2_)_*n*_] − *mE*[GeTe] − *nE*[In_3_SbTe_2_]

where *E* is the DFT total energy of the optimized geometry at zero temperature of the different systems. The resulting formation energies are given in Table 2. The formation energies are all positive which means that the SLs are unstable with respect to phase separation in the parent compounds. However, Δ*H* is smaller than thermal energy under the typical growth conditions which means that the SLs could be formed as a metastable phase during growth.

**Table 2:** Formation energy Δ*H* of the superlattices with respect to the parent compounds (see eqn (1)). The biaxial tensile strain *η* applied on GeTe bilayers (see text) is given in the last column

Superlattice structure	Δ*H* (meV per atom)	*η* (%)
(GeTe)_2_/In_3_SbTe_2_	19.2	2.4
(GeTe)_3_/In_3_SbTe_2_	19.5	2.0
(GeTe)_3_/(In_3_SbTe_2_)_2_	13.5	2.6

The GeTe bilayers are subject to a biaxial strain *η* due to the lattice mismatch with the In_3_SbTe_2_ blocks. The strain *η* is reported in Table 2 for the three SLs as given by *η* = (*a*_SL_ − *a*_GeTe_)/*a*_GeTe_, where *a* is the in-plane lattice parameter of the SL or α-GeTe. The strain is larger in (GeTe)_3_/(In_3_SbTe_2_)_2_ because of the thicker IST block. In all SLs the strain is sizably larger than that of about 0.7% computed for the (GeTe)_2_/Sb_2_Te_3_ SL in ref. 12 because of the larger mismatch in the lattice parameters between bulk GeTe and IST (see Table 1).

This result suggests the possibility to exploit the strain exerted by the IST blocks to reduce the activation barrier for the transformation between the Ferro-GeTe and the Inverted-Petrov configuration of the two GeTe bilayers present in the (GeTe)_3_/(In_3_SbTe_2_)_*n*_ SLs. Before addressing this issue in the next section, we discuss here the local stability of the optimized (GeTe)_3_/In_3_SbTe_2_ SL that we assessed by molecular dynamics (MD) simulations.

We performed MD simulations in a 3 × 3 × 1 supercell of (GeTe)_3_/In_3_SbTe_2_ containing 108 atoms at the equilibrium lattice parameters given in Table 1 for the unit cell. We introduced disorder in the occupation of the Sb/Te sublattice that might be present in SLs as it actually occurs in bulk IST.^42^ To this end, we exchanged four atoms between the two Sb and Te inner layers leading to a concentration of 44/56% of Sb/Te in one plane and the reverse in the other. MD microcanonical simulations 10 ps long were performed at an average temperature of 850 K which is slightly below the melting temperatures of bulk GeTe (998 K)^49^ and IST (907 K).^41^ MD-DFT simulations were performed with the CP2k code,^50^ the PBE functional and Goedecker-type pseudopotentials with three, four, five and six valence electrons for In, Ge, Sb and Te atoms.^51,52^ The KS orbitals were expanded in a triple-zeta-valence plus polarization (TZVP) Gaussian-type basis set and the charge density was expanded in a plane-wave basis set with a cutoff of 100 Ry in order to solve efficiently the Poisson equation within the Quickstep scheme.^50^ BZ integration was restricted to the supercell *Γ* point.

The SL is stable at 850 K as shown by the evolution in time of the *z* position (along the growth axis) of atoms shown in Fig. S1 in the ESI.† No atom interchange among different planes was observed. The simulations were repeated at a higher temperature of 950 K which is still not sufficient to mix different planes on the 10 ps time scale (see Fig. S1 in the ESI†). We then repeated the simulation by expanding the in-plane lattice parameter of the supercell to mimic the strain of 2.6% that is exerted on the (GeTe)_2_ block in the thicker (GeTe)_3_(In_3_SbTe_2_)_2_ SL, still with no interplanar disorder (Fig. S1 in the ESI†).

After having assessed the thermal stability of the (GeTe)_3_/In_3_SbTe_2_ SL, we investigated the switching mechanism between the Ferro-GeTe stable configuration of the SL and the metastable Inverted-Petrov configuration as we describe in the next subsection.

### 3.2 Switching mechanism in the (GeTe)_3_/In_3_SbTe_2_ superlattice

**Fig. 2:**
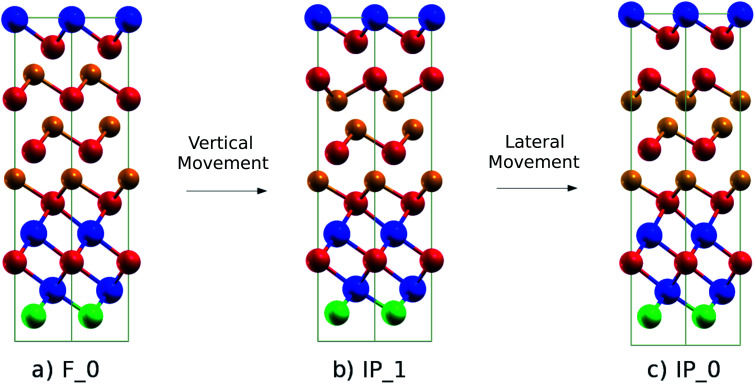
(a) Initial (F_0, Ferro-GeTe), (b) intermediate (IP_1, Switched-Ferro-GeTe) and (c) final (IP_0, Inverted-Petrov) configurations of the RESET transformation. The notation for the different states is the same as that used for the (GeTe)_2_/Sb_2_Te_3_ SL in ref. 10. The color code is the same as that in Fig. 1.

We investigated a possible switching mechanism between the Ferro-GeTe and the Inverted-Petrov configurations of the (GeTe)_2_ sub-block in our (GeTe)_3_/In_3_SbTe_2_ SL by following the path proposed for the (GeTe)_2_/Sb_2_Te_3_ SL in ref. 10. The Inverted-Petrov configuration of the (GeTe)_2_ sub-block is shown in panel (c) of Fig. 2. We follow hereafter the notation of ref. 10 and we call the Ferro-GeTe and Inverted-Petrov configurations F_0 and IP_0. The energy of IP_0 is 0.483 eV per cell (40 meV per atom) higher than that of F_0 once the lattice parameters are fixed to those of F_0 (Ferro-GeTe, see Table 1). After optimizing the lattice parameters as well, the energy of IP_0 is 0.410 eV per cell (34 meV per atom) higher than that of F_0 with relaxed lattice parameters of *a* = 4.233 Å and *c* = 22.284 Å compared to those of F_0 given in Table 1. Since the gain in energy due to the cell relaxation is relatively small, we studied the path for the F_0 → IP_0 transformations by fixing the lattice parameters to those of the ground state F_0 (see Table 1).

As we will see later, the F_0 and IP_0 states can indeed be seen as low resistivity and high resistivity states as they occur in the (GeTe)_2_/Sb_2_Te_3_ SL.^10,53^ The F_0 → IP_0 transformation corresponds to the RESET process while the reverse IP_0 → F_0 is the SET process. On the basis of DFT calculations, both transformations in the (GeTe)_2_/Sb_2_Te_3_ SL were proposed to occur *via* a two-step process.^10^ We follow the same path here for the (GeTe)_3_/In_3_SbTe_2_ SL and the same notation of ref. 10 for the intermediate states along the transformation path.

Regarding the RESET transition, in the first step the Ferro-GeTe (F_0) state transforms into the intermediate IP_1 state (Switched-Ferro state, see Fig. 2b) by means of a vertical movement of a Te and a Ge atomic layer that exchange their positions along the *c* axis. In the IP_1 state the (GeTe)_2_ bilayers turn into a Te–Ge–Ge–Te sequence with BCBA stacking. In the second step, a lateral movement of a Ge and Te plane allows recovering the BCAB stacking and the Te–Ge–Ge–Te sequence of the IP_0 state. A top view of the IP_1 and IP_0 configurations of the GeTe bilayers is shown in Fig. S2 in the ESI.†

**Fig. 3:**
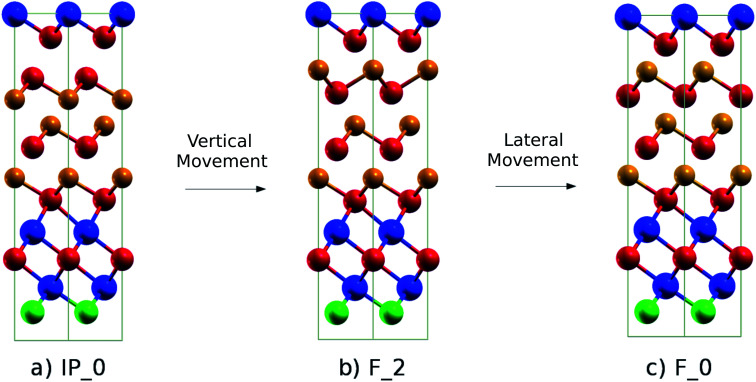
(a) Initial (IP_0, Inverted-Petrov), (b) intermediate (F_2, Switched-Inverted-Petrov) and (c) final (F_0, Ferro-GeTe) configurations of the SET transformation. The notation for the different states is the same as that used for the (GeTe)_2_/Sb_2_Te_3_ SL in ref. 10. The color code is the same as that in Fig. 1.

In the SET process, the IP_0 state transforms into the F_0 state *via* the intermediate state F_2 (Switched-Inverted-Petrov state, see Fig. 3b). The IP_0 → F_2 transformation consists of a vertical movement of a Ge and a Te layer that swaps their positions along the *c* axis giving rise to the Te–Ge–Te–Ge sequence in the BCBA stacking. The final F_0 state is recovered by a lateral movement which turns the Te–Ge–Te–Ge sequence in the BCAB stacking.

We studied the activation barriers for the RESET and SET processes along the transformation path described above by means of NEB-CI simulations as was previously done for the (GeTe)_2_/Sb_2_Te_3_ SL in ref. 10. A word of caution is needed here because the crystal-to-crystal transformation in a real system should proceed *via* nucleation and growth with an activation barrier that should coincide with either the formation free energy of supercritical nuclei or with the activation energy for the growth at the surface of preexisting nuclei. From NEB simulations, we obtain instead the activation barrier for a collective transformation which is an extensive quantity, *i.e.* it depends linearly on the number of cells involved in the transformation. As such the activation barriers obtained by NEB simulations cannot be directly compared with experimental activation energies. The NEB simulations just provide some hints on the path that could be followed during formation and growth of supercritical nuclei and, in our case, on the effect of the strain exerted on the GeTe blocks on the activation barrier.

**Fig. 4:**
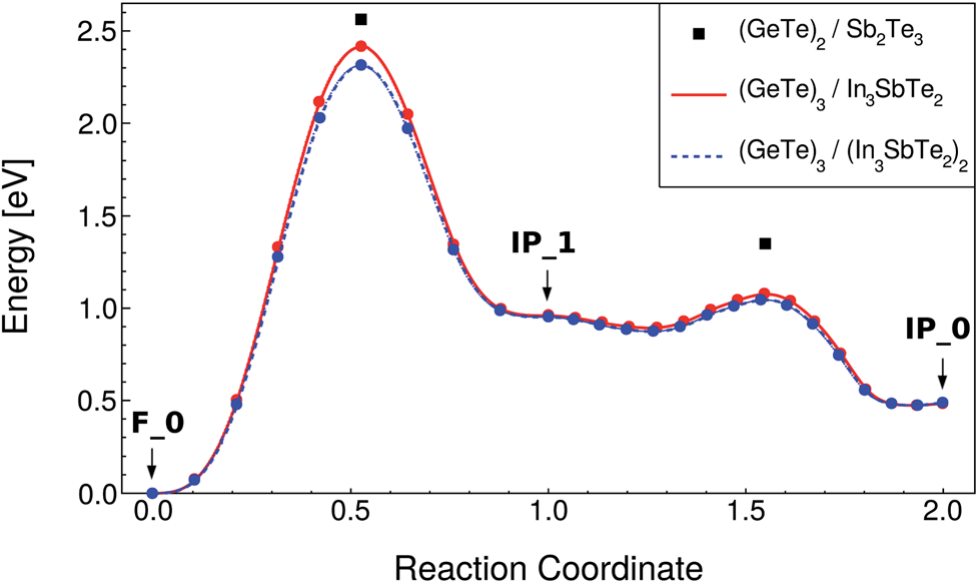
Minimum energy path for the RESET process of the (GeTe)_3_/In_3_SbTe_2_ SL (red line) from NEB-CI simulations along the snake-like pathway (see text). Each point corresponds to an image of the NEB method. The energy of the transition states along the same path for the (GeTe)_2_/Sb_2_Te_3_ SL computed in ref. 10 is reported for the sake of comparison (black squares). The data for the strained system mimicking the (GeTe)_3_/(In_3_SbTe_2_)_2_ SL are also shown (dashed blue line, see text).

**Fig. 5:**
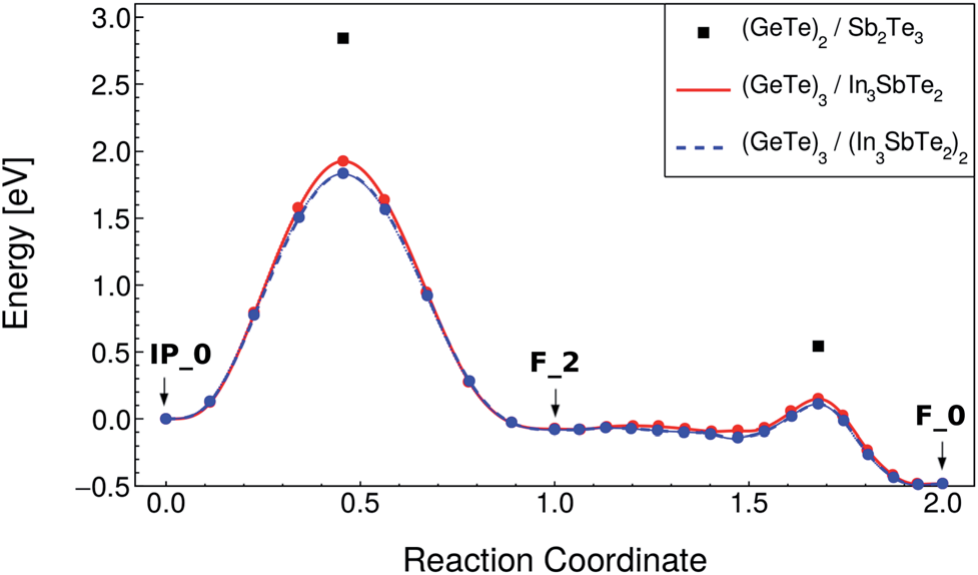
Minimum energy path for the SET process of the (GeTe)_3_/In_3_SbTe_2_ SL (red line) from NEB-CI simulations along the snake-like pathway (see text). Each point corresponds to an image of the NEB method. The energy of the transition states along the same path for the (GeTe)_2_/Sb_2_Te_3_ SL computed in ref. 10 is reported for the sake of comparison (black squares). The data for the strained system mimicking the (GeTe)_3_/(In_3_SbTe_2_)_2_ SL are also shown (dashed blue line, see text).

The NEB calculations were performed by keeping the lattice parameters fixed to the values of the F_0 ground state in both RESET and SET transformations. The NEB calculations were split in two, the first from the initial to the intermediate state, involving a vertical movement, and the second from the intermediate to the final state, involving a lateral movement. The lateral movement can be accomplished along two different pathways according to previous calculations on the (GeTe)_2_/Sb_2_Te_3_ SL: the snake-like and the overhead ones.^10^ These two different pathways are described in ref. 10. The snake-like pathway is the path with the lowest activation energy for both SET and RESET in our SL as it is in the (GeTe)_2_/Sb_2_Te_3_ SL. The resulting energy along the transformation paths for the RESET and SET processes for the snake-like pathway is shown in Fig. 4 and 5. The corresponding plots for the overhead pathways are shown in Fig. S3 and S4 in the ESI.† The highest energy barrier corresponds to the vertical flip while the lowest one corresponds to the lateral movement.

The values for the activation energies (*E*_a_) for the different processes are summarized in Table 3 for the lower energy snake-like pathway. The corresponding values for the overhead pathway are reported in Table S4 in the ESI.† The activation energies for the rate limiting step (vertical movement) for the RESET and SET processes are about 0.15 eV or 0.91 eV lower than the corresponding values calculated for the (GeTe)_2_/Sb_2_Te_3_ SL in ref. 10. The reduction in the activation energy is particularly large for the SET process which suggests that indeed strain engineering can be used to reduce sizably the power consumption in the switching of these SLs. We pursued further this idea by repeating the NEB simulations with strained *a* and *b* lattice parameters of the (GeTe)_3_/In_3_SbTe_2_ SL such as to match the equilibrium lattice parameter of the thicker (GeTe)_3_/(In_3_SbTe_2_)_2_ SL. In this configuration, the (GeTe)_2_ block experiences a strain of about 2.6% equal to that present in the (GeTe)_3_/(In_3_SbTe_2_)_2_ SL with a double IST block (d-IST). The results for the strained SL are also reported in Fig. 4 and 5 and Table 3 for the snake-like pathway. The corresponding results for the overhead pathways are reported in Fig. S3 and S4 and Table S4 in the ESI.† The additional strain leads to a further reduction of the activation barrier of the rate limiting step for the RESET and SET processes of about 0.1 eV. The energies of the initial and intermediate states for the strained SL are compared to those of the unstrained one in Table 4.

**Table 3:** Energy barriers *E*_a_ for the vertical and the lateral snake-like motion of the RESET and SET transitions for the (GeTe)_3_/In_3_SbTe_2_ SL and for the strained system aimed at mimicking the thicker (GeTe)_3_/(In_3_SbTe_2_)_2_ SL (double IST, labeled as “d-IST”). The corresponding results for the (GeTe)_2_/Sb_2_Te_3_ SL (GST) from ref. 10 are reported for the sake of comparison

Memory process	Motion type	Reactant and product	*E*_a_ [eV]
RESET	Vertical flip	F_0 → IP_1	2.41
		(F_0)_d-IST_ → (IP_1)_d-IST_	2.31
		(F_0)_GST_ → (IP_1)_GST_	2.56 (ref. 10)
	Lateral snake-like motion	IP_1 → IP_0	0.12
		(IP_1)_d-IST_ → (IP_0)_d-IST_	0.09
		(IP_1)_GST_ → (IP_0)_GST_	0.39 (ref. 10)
SET	Vertical flip	IP_0 → F_2	1.93
		(IP_0)_d-IST_ → (F_2)_d-IST_	1.83
		(IP_0)_GST_ → (F_2)_GST_	2.84 (ref. 10)
	Lateral snake-like motion	F_2 → F_0	0.22
		(F_2)_d-IST_ → (F_0)_d-IST_	0.19
		(F_2)_GST_ → (F_0)_GST_	0.62 (ref. 10)

**Table 4:** Total energies (per cell) and lattice parameters of the initial, final and intermediate states involved in RESET/SET transitions of the (GeTe)_3_/In_3_SbTe_2_ SL obtained by fixing the lattice parameters to those of the ground state F_0. The configurations labeled as “d-IST” correspond to those of the SL strained in the *ab* plane to mimic the conditions experienced by the (GeTe)_2_ block in the thicker (GeTe)_3_/(In_3_SbTe_2_)_2_ SL (see text). The configurations labeled as “relax” refer to the states in which the lattice parameters have been allowed to relax as well

State	Total energy per cell [eV]	*a* = *b* [Å]	*c* [Å]
F_0	0.00	4.293	21.141
(F_0)_d-IST_	0.005	4.316	21.141
IP_0	0.483	4.293	21.141
(IP_0)_d-IST_	0.492	4.316	21.141
(IP_0)_relax_	0.410	4.233	22.284
IP_1	0.933	4.293	21.141
(IP_1)_d-IST_	0.934	4.316	21.141
(IP_1)_relax_	0.681	4.201	23.262
F_2	0.373	4.293	21.141
(F_2)_d-IST_	0.558	4.316	21.141
(F_2)_relax_	0.317	4.253	21.946

By assuming a linear dependence of the activation barrier on the strain experienced by the (GeTe)_2_ block as was shown to occur in the (GeTe)_2_/Sb_2_Te_3_ SL in ref. 12, we can predict that, for a strain of 3.8% exerted on the (GeTe)_2_ block by an infinitely thick IST block, the activation energy of the rate limiting step is about 2.07 and 1.6 eV for the RESET and SET processes compared with values of 2.56 and 2.84 eV for the (GeTe)_2_/Sb_2_Te_3_ SL (see Table 3).

We have mentioned that the NEB calculations have been performed by fixing the lattice parameters to those of the F_0 state which mimics the conditions that most probably take place in the confined geometry of a phase change memory cell. Nevertheless, we have also assessed the gain in energy of the intermediate IP_1, F_2 and final IP_0 states once the lattice parameters are allowed to relax. The resulting energies reported in Table 4 are only slightly lower than those of the configurations with fixed lattice parameters which indicates that relaxation of the lattice parameters would lead to a minor reduction of the activation energies computed for a fixed cell.

We mention that Non-Equilibrium Green Function (NEGF) calculations based on DFT have recently shown that the activation barriers for the F_0 → IP_0 transitions in the (GeTe)_2_/Sb_2_Te_3_ SL are also only slightly affected by the presence of electric fields and electronic currents.^54^

We recall that a Petrov-like configuration of the (GeTe)_2_ block was also proposed for the low resistance state of the (GeTe)_2_/Sb_2_Te_3_ SL.^55^ We found that this latter configuration is higher in energy by 0.408 eV per cell (34 meV per atom) than that of the Ferro-GeTe state for the (GeTe)_3_/In_3_SbTe_2_ SL and thus it has not been further considered in our analysis. The geometry and the electronic density of states of the Petrov-like state are shown in Fig. S5 in the ESI.†

In summary, a sizable reduction of the activation barriers with respect to the (GeTe)_2_/Sb_2_Te_3_ is predicted for the (GeTe)_3_/IST SLs, especially for the SET process.

### 3.3 Electronic properties of the GeTe/IST superlattices

**Fig. 6:**
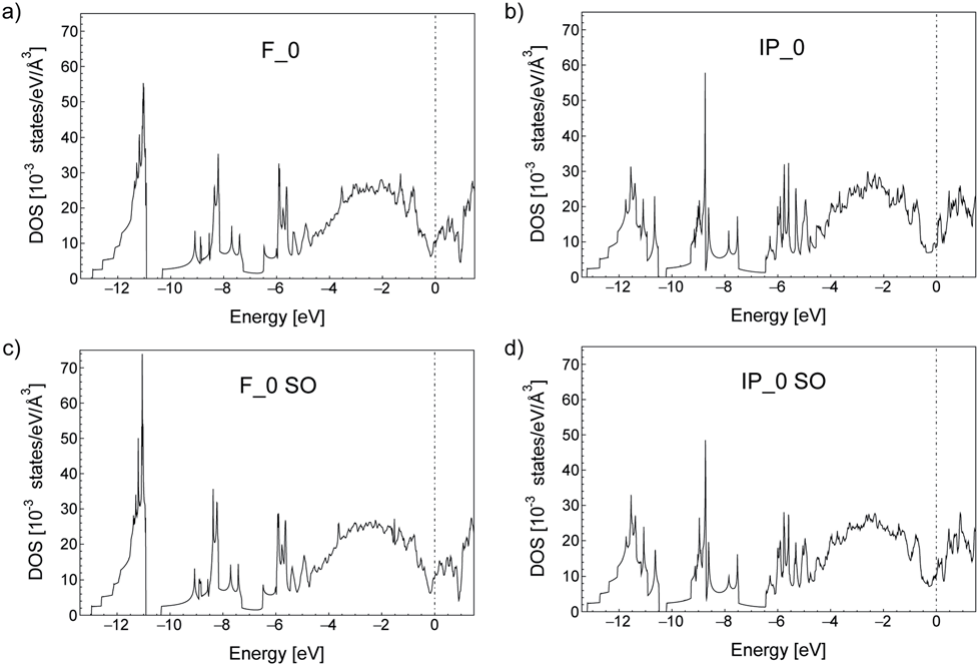
Electronic density of states (DOS) of the (GeTe)_3_/In_3_SbTe_2_ SL in the (a) Ferro-GeTe (F_0) ground state and (b) Inverted-Petrov (IP_0) metastable state. The zero of energy is the Fermi level. The DOS is computed with the tetrahedron method and a 54 × 54 × 18 *k*-point mesh. Spin–orbit interaction is neglected/included in (a and b)/(c and d).

**Fig. 7:**
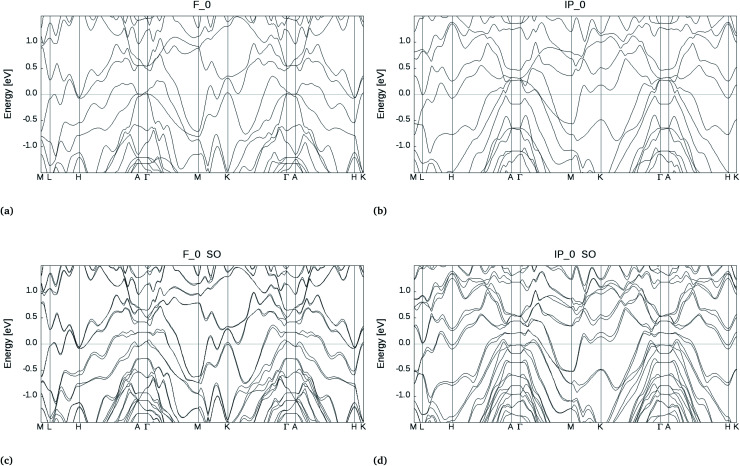
Electronic band structure around the Fermi level (zero of energy) of the (GeTe)_3_/In_3_SbTe_2_ SL in the Ferro-GeTe (F_0) ground state and in the Inverted-Petrov (IP_0) metastable state obtained by neglecting (panels a and b, respectively) or including (panels c and d, respectively) the spin–orbit interaction.

In the bulk, crystalline In_3_SbTe_2_ is metallic with an electronic Density of States (DOS) at the Fermi level of 4.425 × 10^−3^ states per eV per Å^3^ as obtained from DFT calculations.^44^ In contrast, bulk α-GeTe is semiconducting with a DFT-PBE band gap of 0.36 eV (ref. 56 and 57) including spin–orbit interaction. Actually, all three SLs investigated here are metallic as shown in Fig. 6 and S6† with DOSs (without spin–orbit interaction) at the Fermi level of 11.60, 10.77 and 11.33 × 10^−3^ states per eV per Å^3^ for (GeTe)_2_/In_3_SbTe_2_, (GeTe)_3_/In_3_SbTe_2_ and (GeTe)_3_/(In_3_SbTe_2_)_2_ SLs. For the (GeTe)_3_/In_3_SbTe_2_ SL, we report in Fig. 6 the electronic DOS for both the Ferro-GeTe (F_0) ground state and Inverted-Petrov (IP_0) metastable state involved in the switching process. The SL is metallic also in the IP_0 state which should correspond to the high resistivity state with a DOS at the Fermi level of 8.65 × 10^−3^ states per eV per Å^3^ which is only slightly lower than in the F_0 state. However, the electronic band structure is strongly anisotropic as shown in Fig. 7a and b for the F_0 and IP_0 states of the (GeTe)_3_/In_3_SbTe_2_ SL. In particular, in the IP_0 state no bands cross the Fermi level along the *Γ*–*A* direction which is parallel to the *c* axis of the SL (see the Brillouin zone for the hexagonal lattice shown in Fig. 13 of ref. 58). In contrast, in the F_0 state, the Fermi surface cuts the *Γ*–*A* direction as well. The same is true after including the spin–orbit interaction as shown in Fig. 7c and d; the DOS including spin–orbit coupling is shown in Fig. 6c and d. Therefore, the fact that the DOS at the Fermi level is very similar in the two states does rule out the possibility that they might have a markedly different conductivity along the *c* axis of the SL which is the direction actually probed in the device.

To address this issue we have estimated the conductivity tensor *σ*_αβ_ of the F_0 and IP_0 states from the electronic band structure and by assuming an energy-independent relaxation time *τ* as given by2
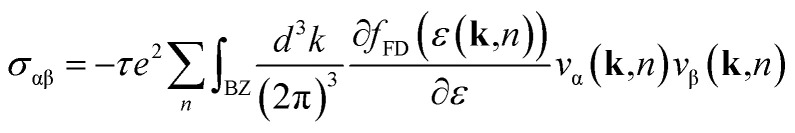
where *e* is the electronic charge, *f*_FD_ is the Fermi–Dirac distribution function evaluated at 300 K, the sum over *n* runs over the electronic bands with energy *ε*(**k**,*n*) and group velocities *v*_α_(**k**,*n*). The calculation of the group velocities and the integration over the BZ in eqn (2) are performed by using a maximally localized Wannier function (MLWF) basis^59,60^ to interpolate first-principles plane-wave results as implemented in the BoltzWann code.^61^ The KS states have been computed on a 10 × 10 × 8 Monkhorst–Pack mesh with the Quantum-Espresso code and the bands have then been interpolated on a denser 600 × 600 × 200 mesh by using an MLWF basis. In the absence of an estimate for the relaxation time, we took the same *τ* for both the low resistivity Ferro-GeTe state (F_0) and the high resistivity Inverted-Petrov state (IP_0) and we simply computed the ratio of the diagonal components of the conductivity tensor which yields *σ*^F_0^_*xx*_/*σ*^IP_0^_*xx*_ = *σ*^F_0^_*yy*_/*σ*^IP_0^_*yy*_ = 1.1(1.0) and *σ*^F_0^_*zz*_/*σ*^IP_0^_*zz*_ = 3.6(3.1); the data in parentheses refer to calculation without spin–orbit interaction. The ratio of the conductivity for the two states is about 3.6 along the *c* axis (*z* component) probed in the device. Although the contrast between the low and high resistivity states might seem small, we remark that a similar value of about 4.1 for the resistance ratio between the F_0 and IP_0 states was found for (GeTe)_2_/Sb_2_Te_3_ SLs by NEGF-DFT simulations.^53^

## 4 Conclusions

By means of DFT calculations we have shown that (GeTe)_3_/In_3_SbTe_2_ superlattices can be devised to display a (GeTe)_2_ block similar to that proposed to be present in (GeTe)_2_/Sb_2_Te_3_ interfacial PCMs.^7,8^ The In_3_SbTe_2_ compound is already known as a phase change material of interest for its high crystallization temperature. The interest for this superlattice structure is twofold. First, the substitution of the Sb_2_Te_3_ block with the In_3_SbTe_2_ one might prevent the incorporation of GeTe bilayers into the Sb-rich blocks as occurs in (GeTe)_*n*_/(Sb_2_Te_3_)_*m*_ superlattices grown by MBE and by other means.^14,17,19,20^ This feature would allow exploiting the crystal-to-crystal phase change proposed for (GeTe)_2_/Sb_2_Te_3_ thus bypassing crystal melting to achieve a substantial power reduction in the RESET process. The crystal-to-crystal transition within the (GeTe)_2_ block in (GeTe)_2_/Sb_2_Te_3_ SLs proposed in the seminal paper of ref. 7 is actually highly debated and doubts have been raised on the very viability of this mechanism because of the difficulties in synthesizing SLs displaying the (GeTe)_2_ blocks that emerged in subsequent studies. The use of IST instead of the Sb_2_Te_3_ block might make the formation of the (GeTe)_2_ block easier.

Secondly, the larger lattice parameter of In_3_SbTe_2_ as compared to Sb_2_Te_3_ permits strain engineering^12^ to be exploited to further reduce the activation barrier for the transformation between the high and low resistivity states of the (GeTe)_2_ block. We have computed the activation barrier for the transformation between the Ferro-like and Inverted-Petrov configurations of the (GeTe)_2_ block along the same two-step path proposed in ref. 10. The activation barrier for the SET process for (GeTe)_3_/In_3_SbTe_2_ is about 0.9 eV lower than that obtained for (GeTe)_2_/Sb_2_Te_3_ within the same framework in ref. 10. This substantial reduction in the activation energy suggests that strain engineering is worth being further explored in iPCMs also exploiting other materials than IST in case the experimental synthesis of (GeTe)_2_/IST SLs turns out to be problematic. In spite of the fact that the system (GeTe)_3_/In_3_SbTe_2_ is metallic in both the high and low resistivity states, we estimate a contrast in resistivity similar to that predicted for (GeTe)_2_/Sb_2_Te_3_ by NEGF methods.^53^ We emphasize again that these conclusions are drawn on the basis of the analysis of the crystal-to-crystal transformation in the (GeTe)_2_ block previously proposed for (GeTe)_2_/Sb_2_Te_3_ SLs. Other mechanisms have been proposed to be effective in (GeTe)_2_/Sb_2_Te_3_ systems grown by MBE and by other means where (GeTe)_2_ is not present, as discussed in the Introduction. We cannot exclude that similar alternative mechanisms might also concur in the switching of (GeTe)_2_/IST SLs investigated here. We hope that these findings will stimulate experimental studies in this direction.

## Conflicts of interest

There are no conflicts of interest to declare.

## Supplementary Material

NA-002-D0NA00577K-s001
