# Supplementary material for: A first-principles study of the switching mechanism in GeTe/InSbTe superlattices
Source: Nanoscale Adv. 2020 Sep 17;2(11):5209–18. doi: 10.1039/d0na00577k (PMC9418462; doi:10.1039/d0na00577k)
Supplement: NA-002-D0NA00577K-s001 [file NA-002-D0NA00577K-s001.pdf]

Electronic Supplementary Material (ESI) for Nanoscale Advances.
This journal is © The Royal Society of Chemistry 2020

A first principles study of the switching mechanism in GeTe/InSbTe superlattices
Electronic Supplementary Information

C. Ribaldone, D. Dragoni, and M. Bernasconi

Dipartimento di Scienza dei Materiali, Università di Milano-Bicocca, Via R. Cozzi 55, I-20125 Milano, Italy

atom	x	y	z	atoms	interplanar distance [Å]	bond length [Å]
In	0	0	-0.0017	In1-Sb1	1.7271	3.0275
Sb	2/3	1/3	0.0310	Sb1-In2	1.6818	3.0019
In	1/3	2/3	0.0628	In2-Te1	1.8349	3.0903
Te	0	0	0.0975	Te1-In3	1.8668	3.1094
In	2/3	1/3	0.1329	In3-Te2	1.9006	3.1297
Te	1/3	2/3	0.1688	Te2-Ge1	1.6449	2.9814
Ge	0	0	0.1999	Ge1-Te3	1.7280	3.0280
Te	2/3	1/3	0.2326	Te3-Ge2	1.6553	2.9872
Ge	1/3	2/3	0.2639	Ge2-Te4	1.7109	3.0183
Te	0	0	0.2963	Te4-In4	1.8696	3.1110
In	2/3	1/3	0.3317	In4-Sb2	1.7271	3.0275
Sb	1/3	2/3	0.3643	Sb2-In5	1.6818	3.0019
In	0	0	0.3962	In5-Te5	1.8349	3.0903
Te	2/3	1/3	0.4309	Te5-In6	1.8668	3.1094
In	1/3	2/3	0.4662	In6-Te6	1.9006	3.1297
Te	0	0	0.5021	Te6-Ge3	1.6449	2.9814
Ge	2/3	1/3	0.5333	Ge3-Te7	1.7280	3.0280
Te	1/3	2/3	0.5660	Te7-Ge4	1.6553	2.9872
Ge	0	0	0.5973	Ge4-Te8	1.7109	3.0183
Te	2/3	1/3	0.6296	Te8-In7	1.8696	3.1110
In	1/3	2/3	0.6650	In7-Sb3	1.7271	3.0275
Sb	0	0	0.6977	Sb3-In8	1.6818	3.0019
In	2/3	1/3	0.7295	In8-Te9	1.8349	3.0903
Te	1/3	2/3	0.7642	Te9-In10	1.8668	3.1094
In	0	0	0.7995	In10-Te10	1.9006	3.1297
Te	2/3	1/3	0.8355	Te10-Ge5	1.6449	2.9814
Ge	1/3	2/3	0.8666	Ge5-Te11	1.7280	3.0280
Te	0	0	0.8993	Te11-Ge6	1.6553	2.9872
Ge	2/3	1/3	0.9306	Ge6-Te12	1.7109	3.0183
Te	1/3	2/3	0.9630	Te12-In1	1.8696	3.1110

TABLE S1: Atomic positions in crystallographic units of the $(GeTe)_2/In_3SbTe_2$ superlattice in the conventional hexagonal cell comprising three formula units. The lattice parameters are given in Table 1 in the article. The interplanar distances and the bond lengths are given in the last two columns.

atom	x	y	z	atoms	interplanar distance [Å]	bond length [Å]
In	0	0	-0.0059	In1-Sb1	1.7459	3.0319
Sb	2/3	1/3	0.0767	Sb1-In2	1.6914	3.0009
In	1/3	2/3	0.1567	In2-Te1	1.8303	3.0813
Te	0	0	0.2433	Te1-In3	1.8816	3.1120
In	2/3	1/3	0.3323	In3-Te2	1.9703	3.1664
Te	1/3	2/3	0.4255	Te2-Ge1	1.5968	2.9486
Ge	0	0	0.5010	Ge1-Te3	1.8124	3.0707
Te	2/3	1/3	0.5867	Te3-Ge2	1.5969	2.9486
Ge	1/3	2/3	0.6623	Ge2-Te4	1.8138	3.0715
Te	0	0	0.7481	Te4-Ge3	1.6005	2.9506
Ge	2/3	1/3	0.8238	Ge3-Te5	1.7851	3.0546
Te	1/3	2/3	0.9082	Te5-In1	1.8160	3.0728

TABLE S2: Atomic positions in crystallographic unit of the $(GeTe)_3/In_3SbTe_2$ superlattice. The lattice parameters are given in Table 1 in the article. The interplanar distances and the bond lengths are given in the last two columns.

atom	x	y	z	atoms	interplanar distance [Å]	bond length [Å]
In	0	0	-0.0063	In1-Sb1	1.7315	3.0344
Sb	2/3	1/3	0.0482	Sb1-In2	1.6638	2.9963
In	1/3	2/3	0.1005	In2-Te1	1.8791	3.1210
Te	0	0	0.1596	Te1-In3	1.8301	3.0918
In	2/3	1/3	0.2172	In3-Te2	1.8350	3.0947
Te	1/3	2/3	0.2749	Te2-In4	1.8664	3.1134
In	0	0	0.3336	In4-Sb2	1.6903	3.0111
Sb	2/3	1/3	0.3867	Sb2-In5	1.7122	3.0234
In	1/3	2/3	0.4406	In5-Te3	1.8233	3.0877
Te	0	0	0.4979	Te3-In6	1.8583	3.1085
In	2/3	1/3	0.5564	In6-Te4	1.9363	3.1558
Te	1/3	2/3	0.6173	Te4-Ge1	1.6067	2.9650
Ge	0	0	0.6678	Ge1-Te5	1.7741	3.0589
Te	2/3	1/3	0.7236	Te5-Ge2	1.6136	2.9687
Ge	1/3	2/3	0.7743	Ge2-Te6	1.7715	3.0574
Te	0	0	0.8301	Te6-Ge3	1.6201	2.9722
Ge	2/3	1/3	0.8810	Ge3-Te7	1.7388	3.0386
Te	1/3	2/3	0.9357	Te7-In1	1.8450	3.1006

TABLE S3: Atomic positions in crystallographic unit of the $(GeTe)_3/(In_3SbTe_2)_2$ superlattice. The lattice parameters are given in Table 1 in the article. The interplanar distances and the bond lengths are given in the last two columns.

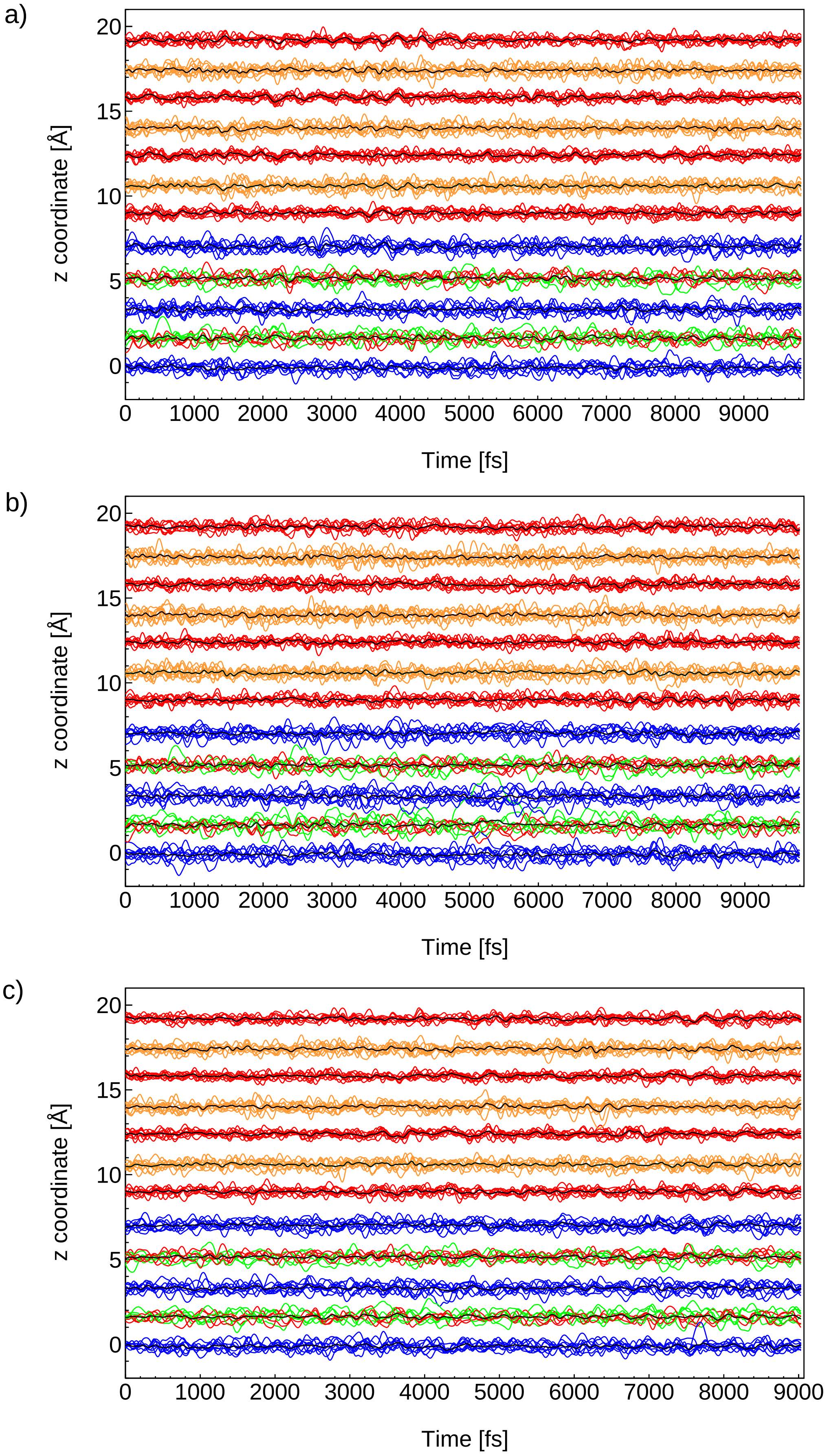

FIG. S1: Atomic positions along the z-axis as a function of time in the molecular dynamics simulation of the $(GeTe)_3/In_3SbTe_2$ superlattice at a) 850 K, b) 950 K, and c) 800 K with a biaxial strain on the $(GeTe)_2$ block of 2.56 % (see article). The color code is the same as in Fig. 1 in the article.

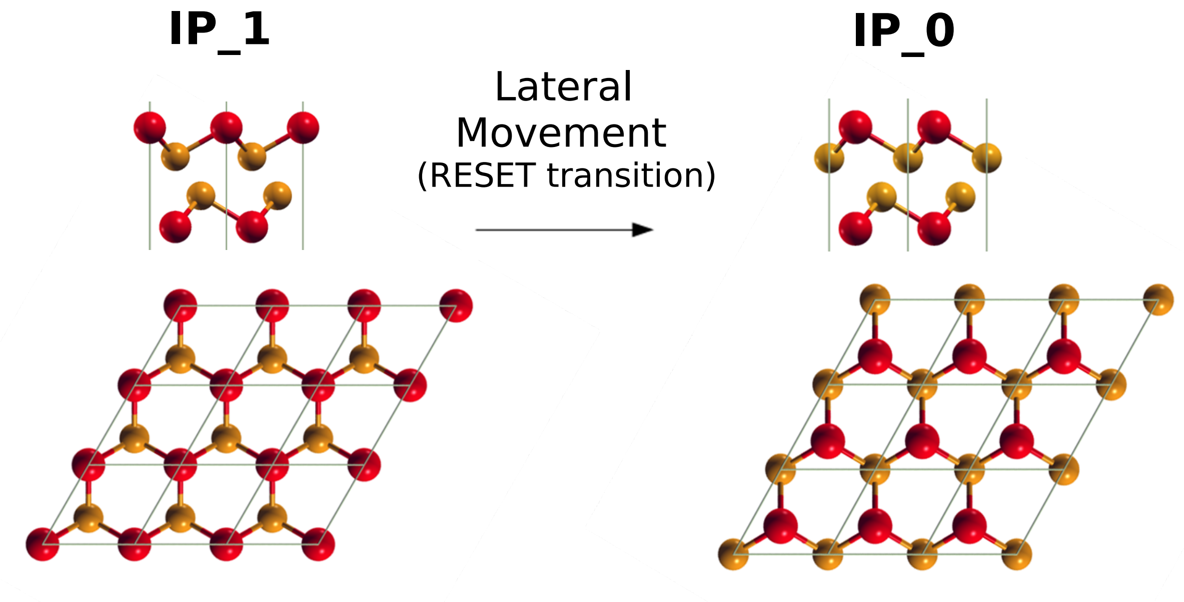

FIG. S2: Lateral movement (second step of the switching RESET process) from the IP_1 to the IP_0 configuration. The upper panels correspond to a side view from the right of the top view reported in the lower panels. Only the GeTe bilayer which plays the fundamental swapping role in the switching process is shown in 3×3 unit cells in the xy plane. Te atoms are red, Ge atoms are orange.

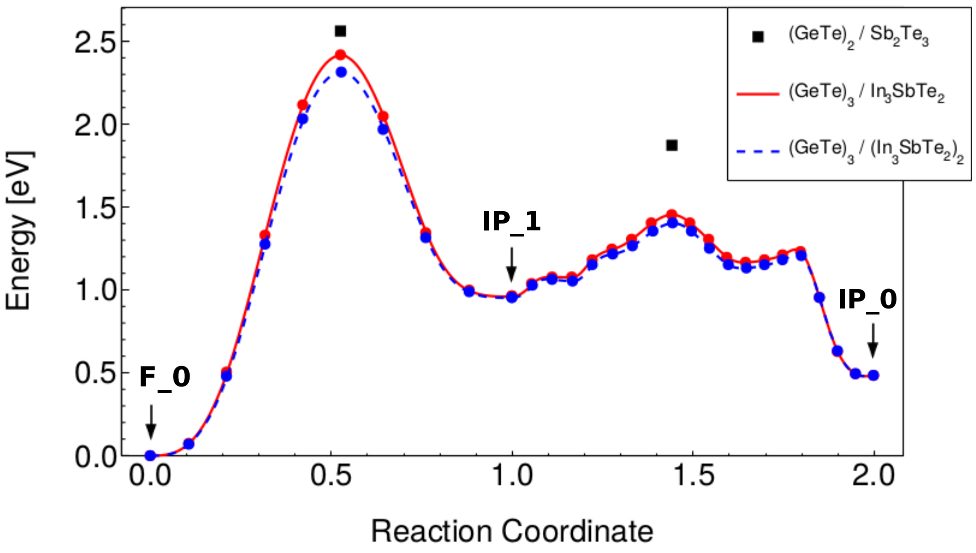

FIG. S3: Minimum energy path for the RESET process of the $(GeTe)_3/In_3SbTe_2$ SL (red line) from NEB-CI simulations along the overhead pathway. Each point corresponds to an image of the NEB method. The energy of the transition states along the same path for the $(GeTe)_2/Sb_2Te_3$ SL computed in Ref.[1] are reported for the sake of comparison (black squares). The data for the strained system mimicking the $(GeTe)_3/(In_3SbTe_2)_2$ SL are also shown (dashed blue line, see article).

Memory Process	Motion type	Reactant and product	Energy barrier [eV]
RESET	Vertical flip	F_0 → IP_1	2.41
		$(F\_0)_{\text{d-IST}} \rightarrow (IP\_1)_{\text{d-IST}}$	2.31
		$(F\_0)_{\text{GST}} \rightarrow (IP\_1)_{\text{GST}}$	2.56[[1]]
	Lateral motion overhead	IP_1→ IP_0	0.49
		$(IP\_1)_{\text{d-IST}} \rightarrow (IP\_0)_{\text{d-IST}}$	0.45
		$(IP\_1)_{\text{GST}} \rightarrow (IP\_0)_{\text{GST}}$	0.92[[1]]
SET	Vertical flip	IP_0 → F_2	1.93
		$(IP\_0)_{\text{d-IST}} \rightarrow (F\_2)_{\text{d-IST}}$	1.83
		$(IP\_0)_{\text{GST}} \rightarrow (F\_2)_{\text{GST}}$	2.84[[1]]
	Lateral motion overhead	F_2 → F_0	0.34
		$(F\_2)_{\text{d-IST}} \rightarrow (F\_0)_{\text{d-IST}}$	0.29
		$(F\_2)_{\text{GST}} \rightarrow (F\_0)_{\text{GST}}$	0.92[[1]]

TABLE S 4: Energy barriers for the vertical and the lateral overhead motion of the RESET and SET transitions for the $(GeTe)_3/In_3SbTe_2$ SL and for strained system aimed at mimicking the thicker $(GeTe)_3/(In_3SbTe_2)_2$ SL (double IST, indicated by d-IST). The corresponding results for the $(GeTe)_2/Sb_2Te_3$ SL (GST) from Ref.[1] are reported for the sake of comparison.

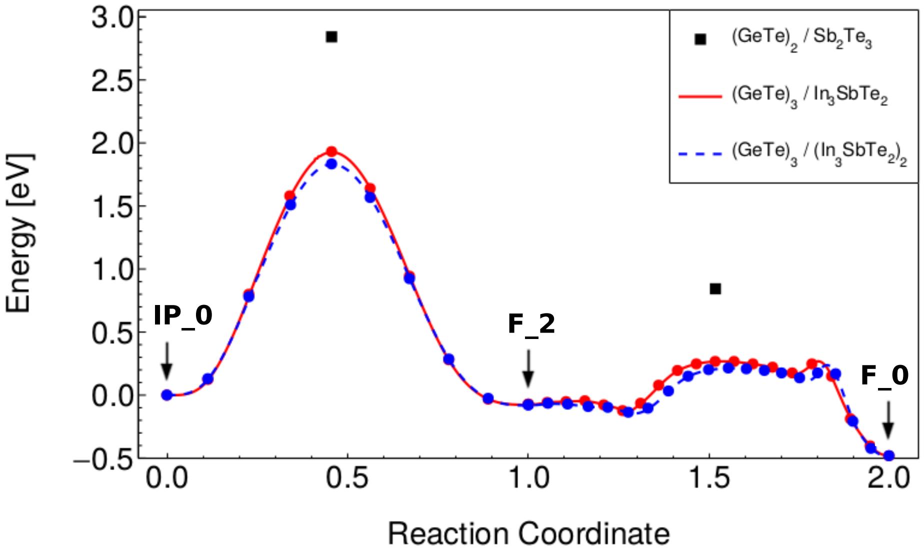

FIG. S4: Minimum energy path for the SET process of the $(GeTe)_3/In_3SbTe_2$ SL (red line) from NEB-CI simulations along the overhead pathway. Each point corresponds to an image of the NEB method. The energy of the transition states along the same path for the $(GeTe)_2/Sb_2Te_3$ SL computed in Ref.[1] are reported for the sake of comparison (black squares). The data for the strained system mimicking the $(GeTe)_3/(In_3SbTe_2)_2$ SL are also shown (dashed blue line, see article).

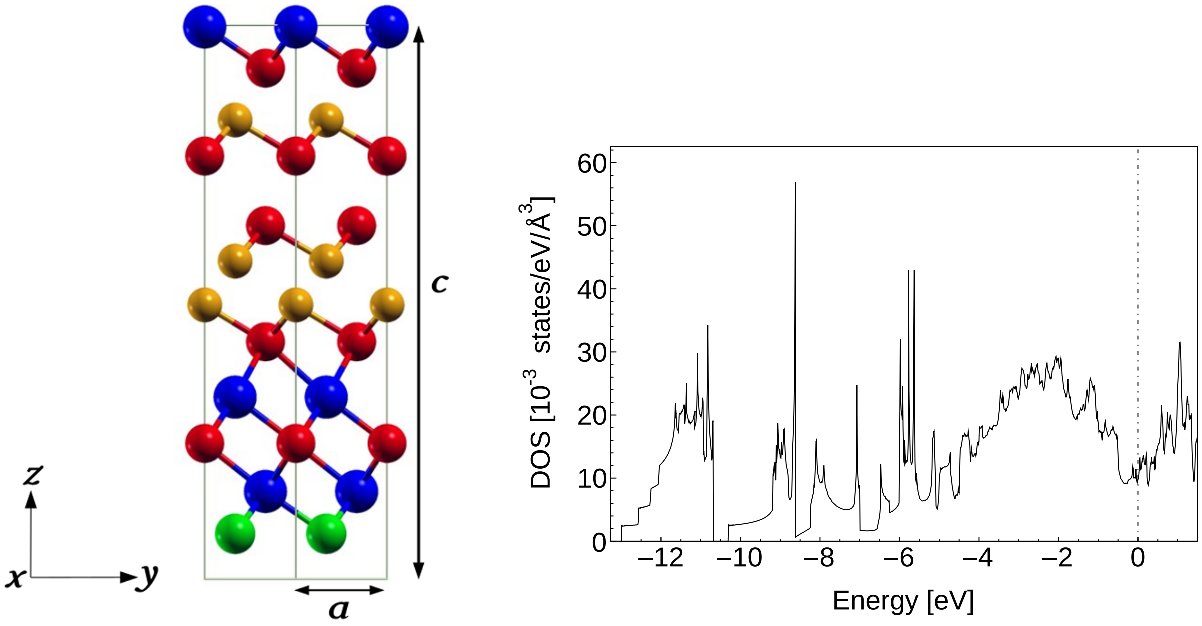

FIG. S 5: (left panel) Geometry of the Petrov-like configuration of the $(GeTe)_2$ block in the $(GeTe)_3/In_3SbTe_2$ SL and (right panel) its electronic density of states (DOS). The optimized equilibrium lattice parameters are a=b=4.233 Å and c=22.284 Å. This configuration is 0.408 eV/cell higher in energy than the Ferro-GeTe (F_0) state (see article). The density of states at the Fermi level (zero of energy) is 9.743 10^{-3} states/eV/Å^3. The DOS is computed with the tetrahedron method and a 54×54×18 k-point mesh.

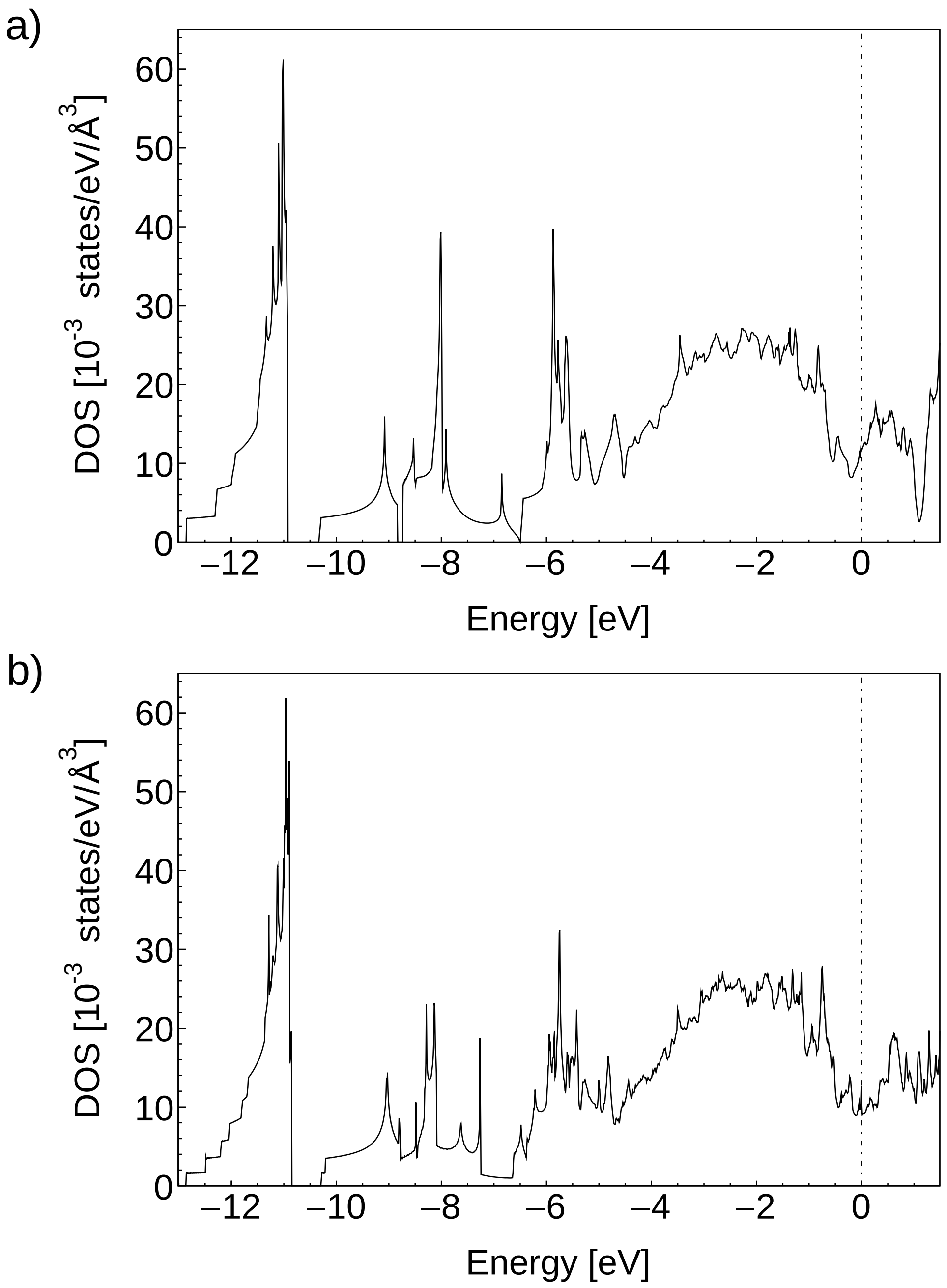

FIG. S6: Electronic density of states (DOS) of the a) $(GeTe)_2/In_3SbTe_2$ and b) $(GeTe)_3/(In_3SbTe_2)_2$ SLs. The zero of energy is the Fermi level. The DOS are computed with the tetrahedron method and a 60×60×60 k-point for the trigonal SL and 54×54×18 k-point mesh for the hexagonal SL. Spin-orbit interaction is neglected.

References

[1] X. Yu and J. Robertson, *Sci. Reports*, 2015, **5**, 12612.
